# *Semiaquilegia quelpaertensis* (Ranunculaceae), a new species from the Republic of Korea

**DOI:** 10.3897/phytokeys.89.21004

**Published:** 2017-11-09

**Authors:** Dong Chan Son, Keum Seon Jeong, Kang-Hyup Lee, Heesoo Kim, Kae Sun Chang

**Affiliations:** 1 Division of Forest Biodiversity and Herbarium, Korea National Arboretum, Pocheon-si 11186, Gyeonggi-do, Republic of Korea

**Keywords:** Ranunculaceae, Hallasan National Park, Eudicots

## Abstract

*Semiaquilegia quelpaertensis* **sp. nov.**, a new species belonging to the family Ranunculaceae, from Hallasan National Park in Jeju-do, Republic of Korea, is described and illustrated. The new species is similar to *Semiaquilegia adoxoides* (DC.) Makino, but can be readily distinguished by a thick underground stem, shallowly lobed leaflets, larger flowers, (4–)6 staminodes and conspicuously rugose tuberculate seed surface.

## Introduction

The genus *Semiaquilegia* Makino is well-known for tuberous-perennial herbaceous plants of the family Ranunculaceae and hitherto consisted of a sole species, *S. adoxoides* (DC.) Makino which is mainly found in China, Korea, Japan and Taiwan (Fu and Orbélia 2001, Hsu et al. 2004, Kadota 2006). Recently, Huang et al. (2017) described *S. guangxiensis* Yan Liu & Y.S. Huang, from the limestone areas of northern Guangxi, China, as a new species of *Semiaquilegia*, with its affinity to *S. adoxoides* and taxonomic implication confirmed by molecular evidence. Additionally, the morphological characteristics, such as the shape of underground stem, the length of pedicel, the shape of petals and the number of stamens and staminodes, have been recently confirmed as key characters in species delimitation within *Semiaquilegia*. As a traditional medicinal plant, *S. adoxoides* is often used against carbuncles, furuncles, swelling, breast carbuncles, scrofula and snakebite; the extract from the underground stem is also helpful for the prevention of cancer (Guang and Wang 2011).

*Semiaquilegia* is currently included in the tribe Isopyreae, subfamily (Kadota, 2006), being widely recognised as closely related to *Aquilegia* L. based on petal evolution (Tucker and Hodges 2005, Damerval and Nadot 2007), geographical affinities (Munz 1946, Nold 2003) and molecular data (Yang et al. 2005). Recently, Wang and Chen (2007) showed that *Semiaquilegia*, *Urophysa* Ulbrich and *Aquilegia* form a monophyletic group, with *Semiaquilegia* as sister to *Aquilegia*. These three genera are characterised by the presence of membranous staminodes, most frequently 5 carpels and spurred petals, but can be distinguished from each other by their habitat, flowering season, flower size, floral structure with the petals divided into lamina and spur, the colour of sepals, the number of staminodia, the presence of spur, seed surface etc. (Munz 1946, Fu and Orbélia 2001, Kadota 2006, Zhao et al. 2016, Erst et al. 2017).

During a recent floristic survey, one species of *Semiaquilegia* which does not appear to be similar to previously reported species was collected at Hallasan National Park in Jeju-do, Republic of Korea. After examination of the various flora and herbarium specimens of Korea and adjacent countries as well as relevant literature (De Candolle 1817, Makino 1902, Wang 1989, Fu and Orbélia 2001, Kadota 2006), it was concluded that the collected specimens represent undescribed species, formally described below as *S. quelpaertensis*.

## Methods

The morphological observations of the new species were conducted based on living plants as well as dry specimens, between 2016 and 2017. The photographs in the field were captured by using a Nikon Coolpix P510 camera. The measurements of the morphological characters were performed by using a digital vernier caliper and data derived from field notes. The flowering and fruiting periods are given as cited on the collector’s labels. Type material has been deposited in the Korea National Arboretum (KH). Voucher specimens of *Semiaquilegia adoxoides* deposited in the KH collection were studied. The conservation status was assessed by applying the IUCN Red List Category criteria (IUCN 2014).

## Taxonomy

### Semiaquilegia quelpaertensis

Taxon classificationPlantaeRanunculalesRanunculaceae

D.C. Son & K. Lee sp. nov.

urn:lsid:ipni.org:names:60475539-2

Figs 1 , 2

#### Diagnosis.

*Semiaquilegia quelpaertensis* is most similar to *S. adoxoides* in general vegetative and floral morphology, but obviously differs from the latter by the shallowly lobed leaflets, larger flowers, (4–)6 staminodes and conspicuously rugose tuberculate seed surface (Table 1).

**Table 1.** Morphological data of *Semiaquilegia* spp.

Species	*S. adoxoides*		*S. guangxiensis*	*S. quelpaertensis*
Source	Makino (1902)	This study	Huang et al. (2017)	This study
Underground stem	thick, 1.5 cm in diameter	thin, 1–2 cm long, 0.3–0.5 cm in diameter	thick, 2–5 cm long, 0.5–2 cm in diameter	thick, 3–5 cm long, 0.6–1 cm in diameter
Basal leaves	ternate; leaf blade suborbicular or reniform, 1–3.5 cm long, 2–4.5 cm wide; segments incised	ternate or biternate; leaf blade ovate, suborbicular or reniform, both length and width 1.2–3.0 cm; segments incised	ternate, rarely biternate; leaf blade ovate to triangular ovate , both length and width 3–9.5 cm; segments shallower lobed	ternate; leaf blade ovate to triangular ovate, both length and width 2–3.5 cm; segments shallower lobed
Flower	4–6.5 mm in diameter	4–6 mm in diameter	15–25 mm in diameter	8–10 mm in diameter
Pedicel	0.3–1.2 cm long	1–2.5 cm long	2.5–12 cm long	0.8–2.5 cm long
Sepals	oblong-lanceolate, 4–6.5 mm long; apex obtuse, but sometimes acute	narrowly elliptic, 4–6 mm long, 1.2–2.5 mm wide; apex acute	broadly elliptic or obovate, 10–20 mm long, 5–10 mm wide; apex rounded or obtuse	narrowly elliptic, 7–8 mm long, 3.0–3.5 mm wide; apex obtuse
Petals	spatulate, 3.0–3.5 mm long, yellow, apex truncate	spatulate, 2.5–3.5 mm long, yellow, base cystic, apex subtruncate, not folded	spatulate, 4–6 mm long, yellow, base tubular, apex retuse, ventrally folded	spatulate, 3.0–3.5 mm long, yellow, base cystic, apex subtruncate, not folded
Androecium	9–14, inserted into petals; staminodes 1–4, half as long as stamen	8–14, inserted into petals; staminodes 2, as long as filaments	20–30, exserted from petals; staminodes ca. 10, 1/2 as long as filaments	16–22, inserted into petals; staminodes (4–)6, 1/2–2/3 as long as filaments
Anther colour	light yellow	yellow	yellow or blackish	yellow
Follicle	9–10 mm long, ca. 3 mm wide	6–7 mm long, ca. 2 mm wide	ca. 10 mm long, ca. 3 mm wide	7–9 mm long, ca. 3 mm wide
Seeds	1.5 mm long, rugose	ca.1 mm long, rugose	ca. 1.5–2.5 mm long, densely rugose	ca.1.5–2.0 mm long, conspicuously rugose

#### Type.

KOREA. Prov. Jeju-do, Jeju-si, Eoseungsaengak, Hallasan National Park, elevation 815 m, 33.4026818°N, 126.4954984°E, 18 April 2017, *K.H. Lee 0300* (holotype KH-1543063!; isotype, 1 sheet, KH-1543065!).

#### Description.

***Herbs*** perennial, 15–25 cm tall. ***Roots*** thin and fibrous. ***Underground stem*** tuberous, oblong, 3–5 cm long, 0.6–1 cm in diameter, light brown. ***Aerial stems*** erect, villose, apically branched. ***Basal leaves*** spirally-alternate, congested on a basal rosette, persistent in mature individuals, several, 1-ternately compound, glabrous, sheathed; petiole 6–12 cm long, villose; leaflets ovate to triangular ovate, 2.0–3.5 cm long, 2.0–3.5 cm wide, 3-parted, segments 2- or 3-lobed. ***Cauline leaves*** spirally-alternate, distributed along the stem, 1–2, shortly petiolate or sessile, similar to basal leaves but smaller. ***Inflorescence*** monochasial cymes, 2–5-flowered; bracts entire, 3-lobed, 3–5 mm long, oblanceolate to obovate; bracteoles 2, 2–3 mm long, oblanceolate. ***Flowers*** actinomorphic, 8–10 mm in diameter, pendulous; pedicel slender, 0.8–2.5 cm long, villose with patent hairs, intermixed with glandular hairs; sepals 5, petaloid, white, usually basally to medially tinged with pink or purple, narrowly elliptic, 7–8 mm long, 3.0–3.5 mm wide, base cuneate, apex obtuse; petals 5, greenish yellow to yellow, spatulate, 3.0–3.5 mm long, apex subtruncate, nectaries cylindrical, shortly spurred; stamens 16–22, filaments 3 mm long, filiform, white, anthers globose, 0.5 mm in diameter, pale yellow; staminodes (4–)6, white, petaloid, membranous, linear-lanceolate, 1/2–2/3 as long as filaments, glabrous; pistils 4–5, glabrous, style ca. 1/6–1/5 as long as ovary, stigma capital. ***Follicles*** 4–5, free, widely divergent, ovoid-oblong, 7–9 mm long, ca. 3 mm wide, apically with a small beak due to the persistent style, striate, striae transversely raised. ***Seeds*** obovoid, 1.5–2.0 mm long, blackish brown, conspicuously rugose tuberculate seed surface.

**Figure 1.**
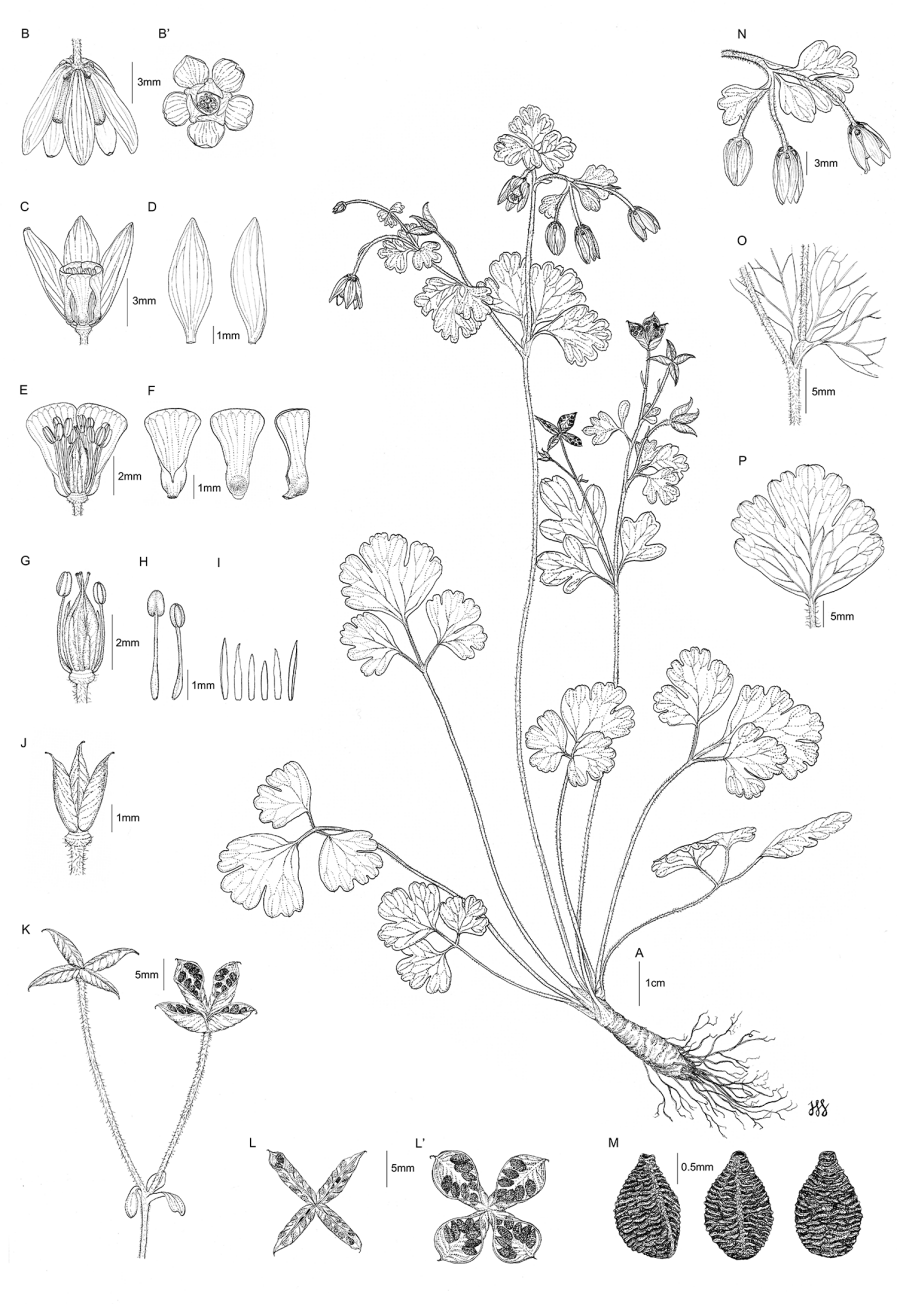
*Semiaquilegia quelpaertensis* D.C. Son & K. Lee, illustrations. **A** Habit **B** Flower (lateral view) **B**’ Flower (top view) **C** Flower with sepals cut away to show petals **D** Sepals **E** Flower with some sepals and petals removed, showing stamens and styles **F** Petals **G** Flower with sepals, petals and some stamens removed, showing stamens, staminodes and pistils **H** Stamens **I** Staminodes **J** Pistils (after fertilisation) **K** Inflorescence in fruiting **L** Follicles (immature) **L**’ Dehisced follicles, showing seed **M** Seed **N** Inflorescence **O** Petiole **P** Leaflet (abaxial surface). Illustrations by Heesoo Kim.

#### Phenology.

Flowering time: April–early May; fruiting time: May.

#### Distribution.

Endemic to the Province Jeju-do (Republic of Korea).

#### Vernacular (Korean) name.

Keun-gae-gu-ri-bal-top (큰개구리발톱; new Korean name).

#### Habitat and ecology.

*Semiaquilegia quelpaertensis* grows in submontane broadleaf forests and in moist valleys at 800–850 m elevation. Its habitat is dominated by *Styrax obassis* Siebold & Zucc. (Styracaceae), with *Dryopteris dickinsii* (Franch. & Sav.) C. Chr. (Dryopteridaceae), *Elatostema umbellatum* (Siebold & Zucc.) Blume (Urticaceae), *Pimpinella hallaisanensis* (W. Lee & G. Jang) G. Jang, W.K. Paik & W. Lee (Apiaceae), *Cardamine tanakae* Franch. & Sav. (Brassicaceae), Peracarpa carnosa var. circaeoides (F. Schmidt ex Miq.) Makino (Campanulaceae), *Viola boissieuana* Makino (Violaceae) and *Anemone stolonifera* Maxim. (Ranunculaceae).

#### Etymology.

The specific epithet of the new species is derived from the type locality, Jeju-do, Republic of Korea.

#### Preliminary conservation status.

Currently, the new species is only known at the type locality and the population size is about 200 mature individuals. It seems that the new species can be ascribed as Endangered (EN) according to the IUCN Red List categories and criteria (IUCN 2014). However, it is possible that further populations could be found in similar habitats of neighbouring areas of Jeju-do, Republic of Korea. Given the current limited field work, this new species could be temporarily considered as Data Deficient (DD).

#### Taxonomic notes.

*Semiaquilegia quelpaertensis* shows morphological similarities with *S. adoxoides* concerning its narrowly elliptic sepals, shorter pedicel and petals subtruncate at apex. Despite these similarities, there are clear differences between these two species, such as the lobed shape of leaflets, the size of the flowers, the surface of seeds and the length and number of staminodes (Table 1), as well as the habitat (mountains for *S. quelpaertensis vs.* lowland for *S. adoxoides*). *Semiaquilegia guangxiensis*, which is endemic to China, also displays shallower lobed leaflets, larger flowers and seeds and more staminodes than *S. adoxoides* (Huang et al. 2017). However, *S. guangxiensis* greatly differs in the 2.5–12 cm long pedicel (*vs.* 0.8–2.5 cm in *S. quelpaertensis*), broadly elliptic or obovate sepals (*vs.* narrowly elliptic sepals in *S. quelpaertensis*) and apex retuse, reflexed along the lower edge, tubular petals (*vs.* apex subtruncate, cylindrical petals in *S. quelpaertensis*; Table 1). Meanwhile, the name *Semiaquilegia dauciformis* D.Q. Wang was proposed by the following characteristics, i.e. underground stem conical, ramose, basal leaves biternate, staminodes 0–6 and the length of style being about half of the ovary or as long as the ovary (Wang 1989). However, *S. dauciformis* have been regarded as a synonym of *S. adoxoides* in Flora of China (Fu and Orbélia 2001). According to the characteristics of the leaves dissection, *S. dauciformis* is similar to *S. quelpaertensis*, but the new species has ternate leaves, larger flowers and staminodes which are 1/2–2/3 of the length of the filaments.

#### Additional specimen examined (paratype).

KOREA. Prov. Jeju-do, Jeju-si, Eoseungsaengak, Hallasan National Park, 17 May 2017, *Lee s.n.* (KH!).

**Figure 2.**
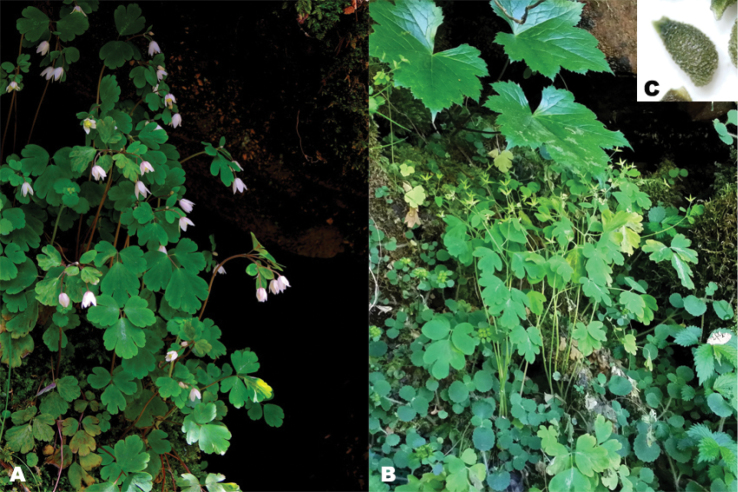
*Semiaquilegia quelpaertensis* D.C. Son & K. Lee, photographs. **A** Flowering plant **B** Fruiting plant **C** Seeds. Photographs by Kang-Hyup Lee.

## Supplementary Material

XML Treatment for Semiaquilegia quelpaertensis
